# High lipoprotein(a) concentrations are associated with lower type 2 diabetes risk in the Chinese Han population: a large retrospective cohort study

**DOI:** 10.1186/s12944-021-01504-x

**Published:** 2021-07-27

**Authors:** Qingan Fu, Lijuan Hu, Yuan Xu, Yingping Yi, Long Jiang

**Affiliations:** 1 Department of Cardiovascular Medicine, the Second Affiliated Hospital of Nanchang University, Nanchang, Jiangxi China; 2 Department of Nursing, Science and Technology College of Jiangxi University of Traditional Chinese Medicine, Nanchang, China; 3 Department of Medical Big Data Center, the Second Affiliated Hospital of Nanchang University, Nanchang, Jiangxi China

## Abstract

**Background:**

Lipoprotein (a) [Lp(a)] is a proven independent risk factor for coronary heart disease. It is also associated with type 2 diabetes mellitus (T2DM). However, the correlation between Lp(a) and T2DM has not been clearly elucidated.

**Methods:**

This was a retrospective cohort study involving 9248 T2DM patients and 18,496 control individuals (1:2 matched). Patients were randomly selected from among inpatients in the Second Affiliated Hospital of Nanchang University between 2006 and 2017. Clinical characteristics were compared between the two groups. Spearman rank-order correlation coefficients were used to evaluate the strength and direction of monotonic associations of serum Lp(a) with other metabolic risk factors. Binary logistic regression analysis was used to establish the correlation between Lp(a) levels and T2DM risk.

**Results:**

The median Lp(a) concentration was lower in T2DM patients than in controls (16.42 vs. 16.88 mg/dL). Based on four quartiles of Lp(a) levels, there was a decrease in T2DM risk from 33.7% (Q1) to 31.96% (Q4) (*P* for trend < 0.0001). Then, Lp(a) levels > 28.72 mg/dL (Q4) were associated with a significantly lower T2DM risk in the unadjusted model [0.924 (0.861, 0.992), *P* = 0.030]. Similar results were obtained in adjusted models 1 [Q4, 0.925 (0.862, 0.993), *P* = 0.031] and 2 [Q4, 0.919 (0.854, 0.990), *P* = 0.026]. Furthermore, in the stratified analysis, Q4 of Lp(a) was associated with a significantly lower T2DM risk among men [0.813 (0.734, 0.900), *P* < 0.001] and those age > 60 years [0.819 (0.737, 0.910), *P* < 0.001]. In contrast, the low-density lipoprotein cholesterol (LDL-C) levels and coronary heart disease (CHD) did not impact these correlations between Lp(a) and diabetes.

**Conclusions:**

There is an inverse association between Lp(a) levels and T2DM risk in the Chinese population. Male patients, especially those aged more than 60 years with Lp(a) > 28.72 mg/dL, are low-risk T2DM individuals, regardless of LDL-C levels and CHD status.

**Supplementary Information:**

The online version contains supplementary material available at 10.1186/s12944-021-01504-x.

## Introduction

Lipoprotein (a) [Lp(a)], discovered in 1963, is plasma-based but has a special structure. It comprises a lipid-rich apolipoprotein (apo) B-containing lipoprotein and apo(a) [1]. The apo(a) gene is highly homologous to the plasminogen gene, and it mainly encodes two tricyclic domains (KIV and KV), and an inactive proteasome domain. Plasma Lp(a) levels are not changed by the environment, but primarily depend on genetic factors, for example, chromosomal mutation of the LPA gene [2]. Elevated Lp(a) levels exert strong proatherogenic and prothrombotic effects contributing to lifelong elevated risks of cardiovascular disease (CVD), stroke, and valvular aortic stenosis [3]. For people with a moderate to a high risk of CVD / coronary heart disease (CHD), Lp(a) should be screened and maintained below 50 mg/dL [4]. The 2018 National Heart, Lung, and Blood Institute (NHLBI) Working Group suggested that Lp(a) > 30 mg/dl (75 nmol/l) or 50 mg/dl (100–125 nmol/l) be considered as “elevated Lp(a)” or “hyperlipoproteinemia(a)”. This implies that, globally, more than 1 billion people have elevated Lp(a) levels and are at a high risk of CVD and aortic stenosis. Guidelines and consensus statements have been published with recommendations for lowering the Lp(a)-mediated risk of CVD [3].

The estimated incidence rates of diabetes and prediabetes among Chinese adults are 11.6% (113.9 million) and 50.1% (493.4 million), respectively, which underscore diabetes as an important public health issue in China [5]. Type 2 diabetes mellitus (T2DM) accounts for most diabetes cases. The global prevalence of diabetes has been on the rise, and is projected to continue increasing in the coming decades [6]. Since T2DM patients are susceptible to CVD, early reports suggested a similar correlation between T2DM and Lp(a) levels. For example, it has been shown that Lp(a) is closely associated with the progression of intima thickness in T2DM [7]. However, some case-control studies have not reported binding conclusions [8–10]. Several large-scale cohort studies have shown an inverse association between T2DM risk and Lp(a) levels, which has not been validated through Mendelian randomization trials [11–13]. A limited number of studies have explored this association in Asian populations, with inconsistent results [14, 15]. Therefore, this study aimed at exploring the association between Lp(a) concentrations and T2DM, and to evaluate potential modification factors in the Chinese population.

## Methods

### Study participants

**Fig. 1:**
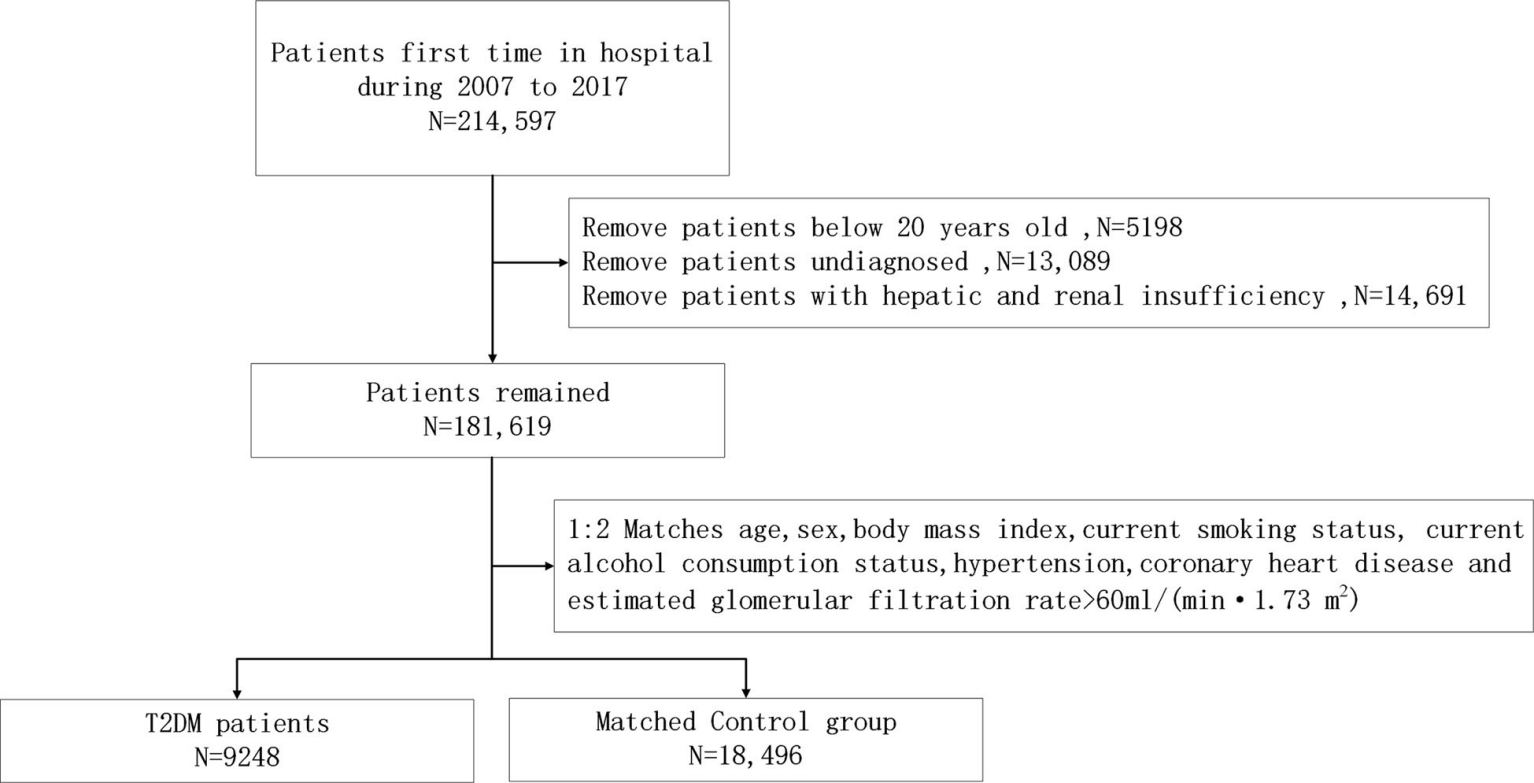
Flow chart of this Study

Electronic medical records of the first hospitalized patients at the Second Affiliated Hospital of Nanchang University between July 2006 and June 2017 were retrospectively reviewed. Notably, 5198 patients aged below 20 years, 13,089 undiagnosed patients, and 14,691 patients with severe liver and renal insufficiency were excluded. Ultimately, a total of 9248 T2DM patients and 18,496 controls were included based on 1:2 matched on sex, age, current smoking status, current alcohol consumption status, body mass index (BMI), CHD, and hypertension following principle of the propensity score matching (PSM) method (Fig. 1). In addition, this study only included patients with an estimated glomerular filtration rate (eGFR) > 60 mL/(min·1.73 m^2^). Ethical approval for this study was obtained from the institutional review board of the Second Affiliated Hospital of Nanchang University, China.

### Clinical and laboratory data

General information, including age, sex, height, weight, smoking, systolic blood pressure (SBP), alcohol consumption, BMI, and diastolic blood pressure (DBP), was recorded. Recorded laboratory data included levels of high-sensitivity C-reactive protein (hsCRP), neutrophil absolute value, total cholesterol (TC), neutrophil-lymphocyte ratio (NLR), prealbumin, low-density lipoprotein cholesterol (LDL-C), platelet-to-lymphocyte ratio (PLR), homocysteine (HCY), uric acid (UA), triglyceride (TG), high-density lipoprotein cholesterol (HDL-c), Lp(a), albumin, absolute value of lymphocytes, glycosylated hemoglobin (HbA1c), fibrinogen, creatinine, eGFR, fasting plasma glucose (FPG), apoA, and apoB.

### Lp(a) measurement

Fresh serum was obtained from patients who had fasted for more than 8 h. Then, the Lp(a) Assay Kit (Latex Enhanced Immunoturbidimetric Method, Beijingantu Inc., China) was used to measure Lp(a) concentrations in human serum. The normal reference range of Lp(a) is from 0 to 3000 mg/dl. Test principle of the method is that Lp(a) reacts with mouse anti-human lipoprotein (a) monoclonal antibody-coated on latex granules and becomes turbid. Measurement of reaction absorbance was recorded at 570 nm and was then correlated with the Lp(a) concentration.

### Definitions of T2DM, hypertension, and CHD

In this study, T2DM was defined according to Classification and Diagnosis of Diabetes: Standards of Medical Care in Diabetes-2021: i. FPG ≥ 126 mg/dL (7.0 mmol/L) after 8 h of fasting. ii. Two hours postprandial blood glucose ≥200 mg/dL (11.1 mmol/L) during the oral glucose tolerance test. Iii. HbA1C ≥ 6.5% [16]. Repeated SBP > 140 mmHg and/or DBP > 90 mmHg (at least 3 times) in the clinic was diagnosed as hypertension [17]. CAD diagnosis was based on coronary angiography (CAG), with confirmation being done if at least one coronary artery or its main branch had diameter stenosis > 50% [18].

### Statistical analysis

PSM is a statistical method for screening treatment and control groups so that the research subjects are comparable in clinical indicators for the purpose of balancing covariates and reducing bias. PSM establishes whether treatment can be used as a response variable and confounding variables as covariates to construct a regression model. The model estimates the propensity score for each object with ranges of between 0 and 1, representing the probability of the subject being classified to the treatment group. Therefore, propensity scores are used to balance the distribution of covariates between the treatment and control groups.

Regarding baseline characteristics, continuous variables are presented as medians (interquartile range [IQR]), and Mann-Whitney U tests were used because the normality test showed that all variables exhibited nonnormality. Categorical variables are presented as frequencies and percentages (%) and were compared using chi-square tests. The Lp(a) levels were divided into four quartiles: quartile 1 (Q1), 0–8.90 mg/dl; quartile 2 (Q2), 8.90–16.74 mg/dl; quartile 3 (Q3), 16.74–28.72 mg/dl; and quartile 4 (Q4), > 28.72 mg/dl. The Spearman rank-order correlation coefficients were used to evaluate the strength and direction of the monotonic associations between Lp(a) and other factors. In addition, the correlation between Lp(a) levels and T2DM was analyzed by binary logistic regression analysis. For these matched case-control data, the odds ratios (ORs) and 95% confidence intervals (95% CIs) were calculated by univariate and multivariate logistic regression models. In model 1, hsCRP and SBP were used in the adjustment; in model 2, creatinine, HCY, TG, LDL-C, HDL-C, plus the factors in model 1 were adjusted for. Furthermore, stratified analyses were conducted by age (≤60 and > 60 years), sex (men and women), LDL-C levels (≤130 mg/dl and > 130 mg/dl), and the presence or absence of CHD. All tests were two sided, with 0.05 as the significance level. R (version 3.6.3), SPSS software version 20.0 (SPSS, Inc., Chicago, Illinois), and Python (version 3.7.) were used to perform all statistical analyses.

## Results

### Clinical characteristics

In total, 9248 T2DM patients and 18,496 controls matched on age, sex, current smoking status, current alcohol consumption, BMI, hypertension, CHD, and eGFR > 60 mL/(min·1.73 m^2^) were randomly selected. Baseline characteristics for the T2DM patients and non-T2DM controls are shown in Table 1. Serum Lp(a) levels were skewed across the 27,744 participants (Figure S1). Briefly, 60 years was the median age after matching. Moreover, the T2DM group exhibited higher TC, LDL-C, and TG levels than the control group. Median Lp(a) levels were lower in the T2DM group than in the control (16.42 vs. 16.88 mg/dl).

**Table 1:** Baseline characteristics of DM and Non-DM cases and controls before and after matching

Characteristics	Before matching		***P***	After matching		***P***
	Control	T2DM		Control	T2DM	
Number	**171,532**	**10,087**		**18,496**	**9248**	
Age, years	60.000 [49.000,70.000]	61.000 [53.000,69.000]	< 0.001	60.0 [52.0,68.0]	60.0 [52.0,68.0]	0.958
Male, n (%)	93,900 (54%)	5325 (52%)	< 0.001	9670 (52%)	4832 (52%)	0.959
Current smoking status, n (%)	113,057 (65%)	7917 (78%)	< 0.001	14,514 (78%)	7262 (78%)	0.918
Current alcohol consumption status, n (%)	9719 (5%)	645 (6%)	0.002	1125 (6%)	594 (6%)	0.267
CHD, n (%)	16,814 (9%)	526 (5%)	< 0.001	904 (4%)	456 (4%)	0.875
Hypertension, n (%)	55,313 (32%)	3916 (38%)	< 0.001	6847 (37%)	3423 (37%)	0.993
BMI	22.769 [21.214,24.347]	23.723 [22.276,25.073]	< 0.001	23.73 [22.27,25.07]	23.74 [22.28,25.10]	0.425
Lp(a), mg/dL	17.270 [9.170,29.720]	16.750 [8.920,28.580]	0.001	16.88 [8.92,29.07]	16.42 [8.8,28.0]	0.015
SBP, mmHg	126.000 [119.660,136.000]	130.000 [120.000,140.000]	< 0.001	127.83 [120.0,138.0]	129.91 [120.0,139.25]	< 0.001
DBP, mmHg	76.000 [70.000,80.000]	77.310 [72.680,81.000]	< 0.001	76.54 [70.0,80.92]	77.3 [72.83,81.0]	< 0.001
TC, mmol/L	4.290 [3.620,5.000]	4.520 [3.840,5.250]	< 0.001	4.46 [3.77,5.16]	4.53 [3.86,5.25]	< 0.001
TG, mmol/L	1.190 [0.870,1.710]	1.420 [1.010,2.070]	< 0.001	1.34 [0.96,1.93]	1.41 [1.0,2.07]	< 0.001
HDL-c, mmol/L	1.050 [0.860,1.270]	1.010 [0.840,1.210]	< 0.001	1.04 [0.86,1.25]	1.01 [0.85,1.22]	< 0.001
LDL-c, mmol/L	2.520 [1.990,3.110]	2.690 [2.120,3.300]	< 0.001	2.64 [2.09,3.22]	2.7 [2.14,3.3]	< 0.001
ApoA, mmol/L	1.050 [0.880,1.230]	1.050 [0.900,1.220]	0.081	1.06 [0.9,1.24]	1.05 [0.9,1.22]	0.096
ApoB, mmol/L	0.770 [0.640,0.930]	0.830 [0.690,1.000]	< 0.001	0.81 [0.67,0.96]	0.83 [0.69,1.0]	< 0.001
eGFR, mL/(min·1.73 m^2^)	97.483 [81.653,113.860]	101.990 [83.391,120.894]	< 0.001	97.81 [85.03,112.20]	104.85 [88.69,122.85]	< 0.001
Fasting Glucose, mmol/L	4.990 [4.510,5.740]	7.820 [5.940,11.030]	< 0.001	5.11 [4.62,5.87]	7.88 [5.99,11.03]	< 0.001
HbA1c, %	5.713 [5.500,6.031]	7.700 [6.500,9.800]	< 0.001	5.79 [5.54,6.10]	7.7 [6.5,9.86]	< 0.001
Creatinine, mmol/L	68.879 [57.200,82.610]	65.800 [54.080,80.200]	< 0.001	67.4 [56.9,79.0]	63.96 [52.9,75.6]	< 0.001
HCY, μmol/L	13.447 [11.133,16.380]	11.842 [9.910,14.452]	< 0.001	13.090 [11.10,15.60]	11.57 [9.76,13.82]	< 0.001
hsCRP, mg/dL	4.362 [2.000,15.492]	4.186 [2.365,10.414]	0.628	3.85 [2.04,11.53]	3.917 [2.3,9.03]	0.317

Continuous variables presented as median (25, 75%)

**ApoA,** apolipoprotein A; **ApoB,** apolipoprotein B; **BMI,** body mass index; **CHD,** coronary heart disease; **DBP,** diastolic blood pressure; **eGFR,** estimated glomerular filtration rate; **HbA1c,** glycosylated hemoglobin; **hsCRP,** high-sensitivity C-reactive protein; **HCY,** homocysteine; **HDL-c,** high density lipoprotein cholesterol; **LDL-c,** low density lipoprotein cholesterol; **Lp(a),** lipoprotein (a); **SBP,** systolic blood pressure; **TC,** total cholesterol; **TG,** triglyceride

### Correlations between Lp(a) concentrations and other clinical profiles

Levels of Lp(a) exhibited the following: i. significant but very weak positive correlations with most indices, such as LDL-C (r: 0.185), TC (r: 0.113), and apoB (r: 0.136) (Table S1); ii. significant but very weak negative correlations with TG (r: − 0.073), albumin (r: − 0.027), eGFR (r: -0.050), HCY (r: − 0.021), and fasting glucose (r: − 0.037) (Table S1); iii. Significantly positive correlations with CHD (r: 0.019), ischemic stroke (r: 0.047), and hypertension (r: 0.018); iv. Negative correlations with T2DM (r: − 0.012) and hypertriglyceridemia (r: − 0.097) (Table S1); v. significant positive correlations with inflammatory markers, such as hsCRP (r: 0.053), NLR (r: 0.054), and PLR (r: 0.111).

### Lp(a) and T2DM risk

**Fig. 2:**
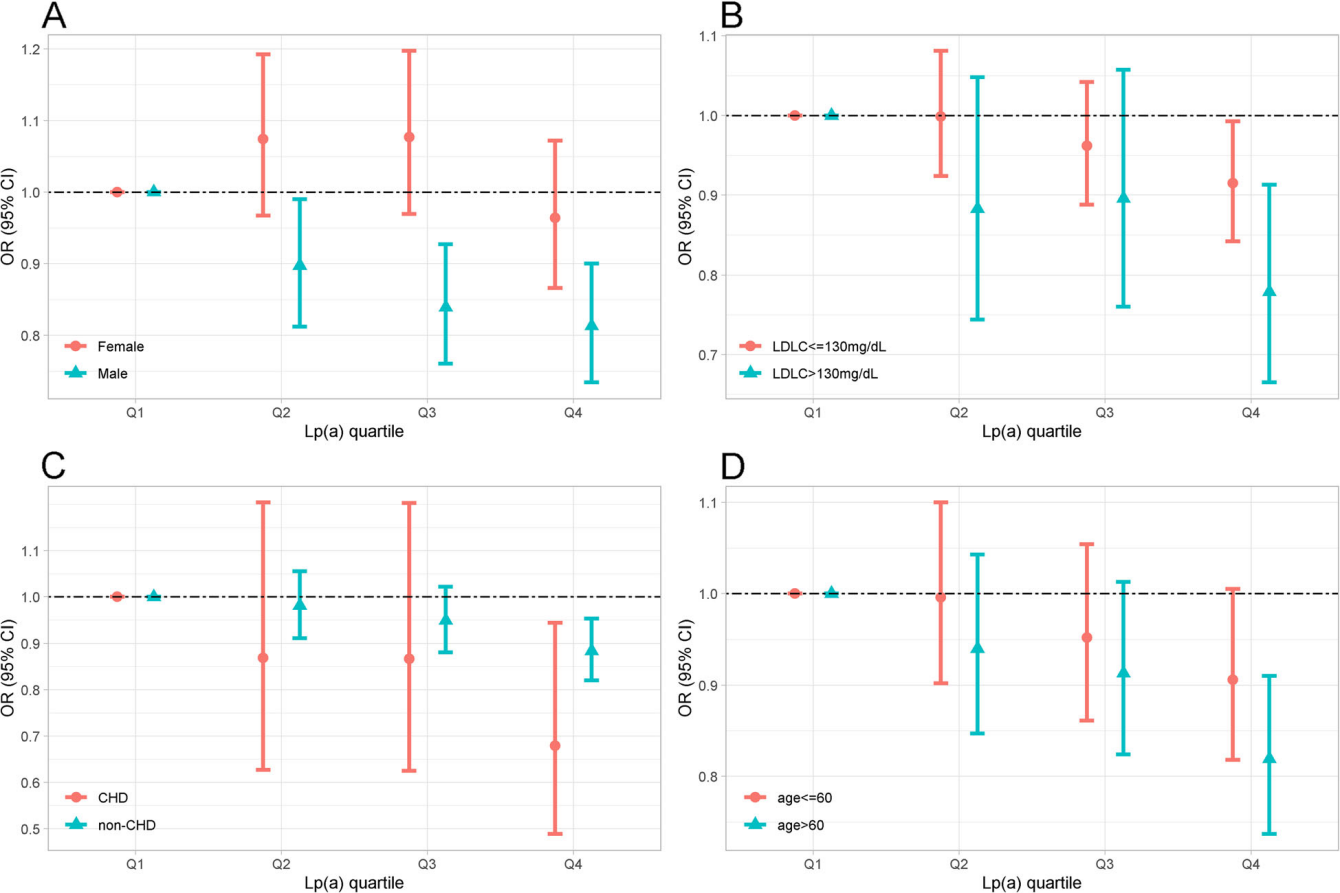
Odd ratios (95% confidence intervals) for T2DM of Lipoprotein (a) quartiles stratified by sex (**A**), LDL-C(**B**), CHD(**C**) and age (**D**). The model of A was adjusted for LDL-C, creatinine, hsCRP, SBP, HCY. The model of B was adjusted for creatinine, hsCRP, SBP, HCY. The model of C was adjusted for LDL-C, creatinine, hsCRP, SBP, HCY. The model of D was adjusted for LDL-C, creatinine, hsCRP, SBP, HCY

Based on the four Lp(a) quartiles, a decreasing trend in the incidence of T2DM was found, from 33.7% (Q1) to 31.96% (Q4) (*P* for trend < 0.0001) (Figure S2A), and an increasing trend in the incidence of CHD from 4.57% (Q1) to 5.47% (Q4) (Figure S2B). A decreasing trend in the incidence of T2DM combined T2DM with CHD was found, from 1.74% (Q1) to 1.54% (Q4) (Figure S2C). A binary conditional logistic regression model was used to determine whether Lp(a) concentrations were independently associated with the onset of T2DM (Table 2). Using an unadjusted model, the ORs and 95% CIs for Lp(a) quartiles 2–4 were found to be 1.020 (0.951, 1.094), 0.993 (0.925, 1.065), and 0.924 (0.861, 0.992), respectively. After adjusting for hsCRP and SBP in model 1, similar results to those in the unadjusted group [Q4, 0.925 (0.862, 0.993), *P* = 0.031] were obtained. Furthermore, after adjusting for the factors included in model 1 plus creatinine, homocysteine, TG, LDL-C, and HDL-C (Table 2), similar results to model 1 were obtained as follows: Lp(a) was significantly associated with T2DM risk with ORs and 95% CI in the Q4 group [0.919 (0.854, 0.990), *P* = 0.026]. In the stratified analysis, Q4 of Lp(a) had a significantly lower T2DM risk in men [0.813 (0.734, 0.900), *P* < 0.001] (Fig. 2A) and the elderly population aged > 60 years [0.819 (0.737, 0.910), *P* < 0.001] (Fig. 2D). Conversely, LDL-C levels and CHD had no impact on the correlation between Lp(a) and T2DM (Fig. 2B and C).

**Table 2:** Odd ratios (95% confidence intervals) for T2DM and Lp(a) concentration

Group	Unadjusted model		Model1		Model2	
	OR (95% CI)	*P* value	OR (95% CI)	*P* value	OR (95% CI)	*P* value
Q1	Reference		Reference		Reference	
Q2	1.020 [0.951, 1.094]	0.580	1.013 [0.944, 1.087]	0.712	1.009 [0.939, 1.085]	0.798
Q3	0.993 [0.925,1.065]	0.835	0.987 [0.920,1.059]	0.710	0.981 [0.912,1.055]	0.597
Q4	0.924 [0.861,0.992]	0.030	0.925 [0.862,0.993]	0.031	0.919 [0.854,0.990]	0.026
Per Q effect	0.974 [0.953,0.996]		0.974 [0.953,0.996]		0.969 [0.947,0.992]	
*P* for trend		0.022		0.023		0.009

Model1: adjusted for high-sensitivity C-reactive protein, systolic blood pressure

Model 2: adjusted for model 1 plus, creatinine, homocysteine, low density lipoprotein, high density lipoprotein, triglyceride

The 25th, 50th, and 75th percentile cut-off points for corresponding Lp(a) are 8.90, 16.74, and 28.72 mg/dL, respectively

## Discussion

This study found that, at Lp(a) concentrations > 28.72 mg/dL, there was a significant inverse correlation between Lp(a) concentrations and T2DM in the Chinese population. However, this was not the case in all populations. In particular, there was a significant inverse association between elevated Lp(a) levels and T2DM only in men and individuals aged above 60 years, and these relationships were not impacted by LDL-C levels and CHD status.

Studies on the relationship between Lp(a) levels and diabetes have not established conclusive findings [9, 19–22]. For instance, Wang et al. found that Lp(a) levels in diabetic patients was significantly higher than those in nondiabetic controls in the Chinese population [21]. In contrast, another prospective cohort study by Muhanhali et al. found that Q1 of Lp(a) levels (median 8.44 mg/dL) had a 71% higher risk of T2DM compared to the overall Lp(a) median level of 74.93 mg/dL [23]. Other studies have reported no correlation between Lp(a) levels and T2DM. Liu et al. found that this association was evident at some Lp(a) concentration range but that the relationships could be attributed to chance and not an actual association [14]. Elsewhere, a Mendelian randomization study revealed that low Lp(a) levels do not have causal correlation with diabetes. However, a causal relationship between large Lp(a) subtype size and T2DM was found [12]. This study found that elevated Lp(a) levels (> 28.72 mg/dL) potentially lowered T2DM risk, but only in men and those aged above 60 years. In tandem with findings in this study, a recent study found that the prevalence and incidence rates of T2DM increased with decreasing baseline levels of Lp(a) [24].

**Fig. 3:**
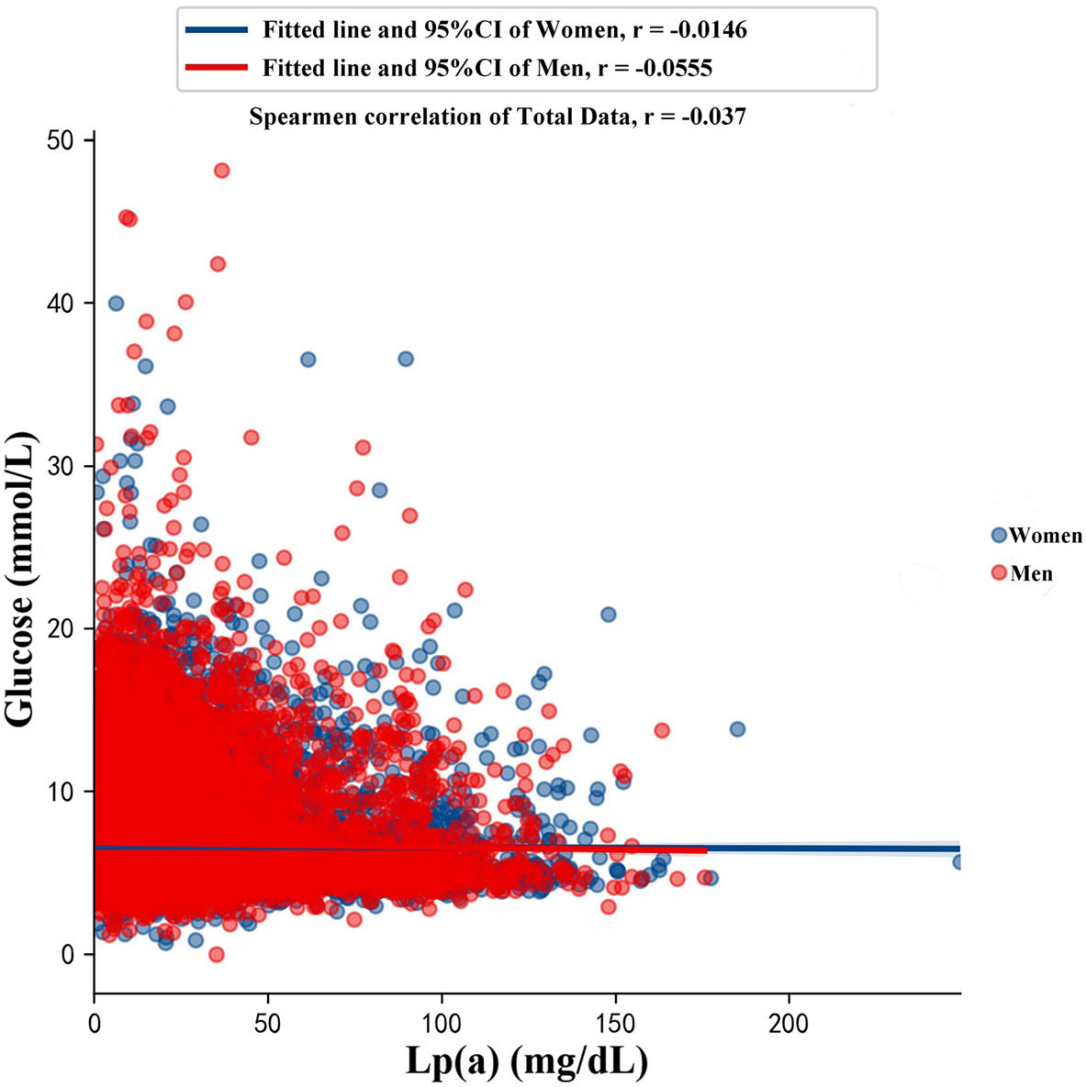
Simple scatter plot and fit line with 95%(CI) of the correlation coefficent in Spearman correlation of Lp(a) and glucose

This study also found a significant inverse relationship between elevated Lp(a) levels and T2DM in men and in individuals aged over 60 years. These results implied that not all populations exhibit an inverse relationship between elevated Lp(a) and T2DM risk. Similar findings were reported by Boronat et al. who found a significant reduction in the risk of age-related diabetes at Lp(a) > 46 mg/dL [25]. Furthermore, in this study, men with T2DM exhibited lower Lp(a) levels than women (15.97 vs. 16.94 mg/dL, respectively, *P* = 0.001)); however, only men exhibited a significant negative correlation between Lp(a) and glucose levels (men, − 0.0555 *P* < 0.001; Woman r: -0.0146, *P* = 0.0919) (Fig. 3). In contrast, in the nondiabetic population, there was no significant difference in median Lp(a) levels between men and women (16.0 vs. 16.82 mg/dL, *P* = 0.246). The findings in this study are consistent with those from a previous study performed at Tehran Medical University [22], which found that women with T2DM had increased Lp(a) levels regardless of menopausal status. A Korean study [26] also found that women had significantly higher Lp(a) levels when compared to than men (median: 13.2 vs. 10.6 mg/dL). These differences could be attributed to various factors, such as ethnic differences associated with genetic differences that may impact Lp(a) levels. Hungarian individuals were found to have lower median plasma levels than Tyrolean individuals (8.3 vs. 14.1 mg/dL), whereas Chinese individuals had lower median plasma levels than Sudanese individuals (7.2 vs. 45.7 mg/dL). In Asian populations, significant heterogeneity exists. The median Lp(a) level in Indian men was found to be significantly higher than that in Chinese men (35 vs. 19 mg/dL, *P* = 0.0001). Among women, there were no statistically significant differences between Indian and Chinese individuals (41 mg/dl vs. 27 mg/dl, *P* = 0.1642) [27, 28]. Factors that potentially influence Lp(a) levels, such as insulin therapy alone, may also elevate Lp(a) levels, which were found to be twice as high in non-right-handed patients as in right-handed patients [29, 30]. Therefore, differences in age and sex should be considered in actual clinical practice. This study found that elevated Lp(a) levels lowered the risk of T2DM. Future large well-designed prospective cohort studies are needed to validate these results.

The mechanisms through which serum Lp(a) levels are associated with diabetes risk remain elusive. Generally, studies on inverse relationships have reported that Lp(a) levels are inversely correlated with insulin resistance. A Korean study [31] revealed that Lp(a) levels were significantly negatively associated with fasting insulin levels, insulin resistance (IR), and insulin secretion (IS). Among patients with low Lp(a) levels, a significantly higher risk of diabetes development was reported in patients with IR alone than in patients with IS impairment alone. A similar conclusion was found in another study that assessed the Turkish population [32]. Moreover, T2DM individuals present with hyperinsulinemia induced by peripheral insulin tolerance. Since elevated insulin concentrations impede the production of posttranscriptional apoA in liver cells, plasma Lp(a) levels are consequently suppressed [33]. However, the specific mechanisms should be further elucidated.

Inconsistencies in measurement methods can result in differences in mean serum Lp(a) levels. This potentially explains why associations between Lp(a) and T2DM vary among studies. Various drugs have an effect on Lp(a) levels. Proprotein convertase subtilisin/kexin type 9 (PCSK9) inhibitors have been confirmed to reduce Lp(a) levels [34]. Treatment with extended-release nicotinic acid has been significantly associated with suppressed Lp(a) levels [35]. Other non-lipid lowering drugs, such as raloxifene have also been shown to have an effect on Lp(a) levels. Oral raloxifene treatment significantly reduced plasma Lp(a) levels, especially in postmenopausal women [36]. In addition, some natural products affect plasma Lp(a) levels. A positive clinical significance of flaxseed supplementation for patients whose plasma Lp(a) levels remained high after statin therapy has been reported [37]. Unfortunately, the database used in the present study did not contain data on drugs used by the included patients. Future studies should aim to confirm whether drugs that influence Lp(a) levels can impact the relationship between Lp(a) and T2DM.

### Study strength and limitations

This is a large-scale, well-matched retrospective cohort study on the relationships between Lp(a) concentration and T2DM in a Chinese population. It shows that there is an inverse association. In addition, this effect was significant only in aged patients (> 60 years old) and men, and this relationship was not altered by LDL-C levels and CHD status. However, there are some limitations to this study. First, as a retrospective study, This study could only assess the correlation between Lp(a) levels and T2DM risk but not the causal relationship. Second, the data used in this study were based on hospital and clinical patients, which may have introduced residual confounding factors. Regression study designs are easily influenced by such confounding factors, which may exaggerate or weaken the correlation of existing variables. To reduce the influence of confounding factors, definite diabetic risk factors (excluding multicollinearity among covariates) were incorporated into logistic models for stratified analysis in this study. Third, recent studies have reported that Lp(a) levels are likely to be affected by statins [38], but the data on whether this study participants were taking lipid-lowering drugs or had been treated with some natural products was missing, which may have introduced a bias into this study.

## Conclusion

In conclusion, there is an inverse association between Lp(a) levels and T2DM risk. This effect is significant only in aged patients (> 60 years old) and men. Such an inverse correlation provides some evidence for a deeper understanding of Lp(a) in diabetic patients. Therefore, male patients who are more than 60 years of age with Lp(a) levels greater than 28.72 mg/dL should be considered to be less at risk of T2DM, regardless of LDL-C levels and CHD status.

## Supplementary Information

**Additional file 1.**

## Data Availability

The datasets used and/or analyzed during the current study available from the corresponding author on reasonable request.

## Abbreviations

apoA: apolipoprotein A
apoB: apolipoprotein B
BMI: Body mass index
CAG: Coronary angiography
CVD: Cardiovascular disease
CHD: Coronary heart disease
hsCRP: high-sensitivity C-reactive protein
DBP: Diastolic blood pressure
eGFR: glomerular filtration rate
GLU: Glucose
HbA1c: Glycosylated hemoglobin
HCY: Homocysteine
HDL:-c: High density lipoprotein cholesterol
IQR: Interquartile range
IR: Insulin resistance
IS: Insulin secretion
LDL-C: low density lipoprotein cholesterol
Lp(a): Lipoprotein (a)
NHLBI: National Heart, Lung, and Blood Institute
NLR: Neutrophil-Lymphocyte Ratio
PCSK9: Proprotein convertase subtilisin/kexin type 9
PLR: Platelet-to-lymphocyte ratio
PSM: Propensity Score Matching
SBP: Systolic blood pressure
TC: Total cholesterol
TG: Triglyceride
T2DM: Type 2 diabetes mellitus
UA: Uric acid
