# Supplementary material for: High lipoprotein(a) concentrations are associated with lower type 2 diabetes risk in the Chinese Han population: a large retrospective cohort study
Source: Lipids Health Dis. 2021 Jul 27;20:76. doi: 10.1186/s12944-021-01504-x (PMC8317300; doi:10.1186/s12944-021-01504-x)
Supplement: Supplementary file 1 — Additional file 1. [file 12944_2021_1504_MOESM1_ESM.docx]

**High lipoprotein(a) concentrations are associated with lower type 2 diabetes risk in the Chinese Han population: A large** **retrospective cohort study**

Qingan Fu^1^, Lijuan Hu^2^, Yuan Xu^3^_,_ Yingping Yi^3^, Long Jiang^1^

**Supplements**

**Supplemental Table S1 Correlation between lipoprotein (a) and the variables**

**Supplemental Figure S1 Serum lipoprotein(a) level distribution of participants**

**Figure S2 Prevalence of T2DM and CHD according to quartiles of Lp(a) (mg/dl).**

**Table S1 Correlation between lipoprotein (a) and the variables**

|  | **r-cofe** | ***P*-value** |
| --- | --- | --- |
| **Sex** | -0.015 | 0.015 |
| **Age** | 0.057 | <0.001 |
| **T2DM** | -0.012 | 0.040 |
| **Hypertension** | 0.018 | 0.003 |
| **CHD** | 0.019 | 0.002 |
| **Atrial fibrillation** | -0.002 | 0.681 |
| **Ischemic stroke** | 0.047 | <0.001 |
| **Hemorrhagic stroke** | -0.003 | 0.59 |
| **Hypertriglyceridemia** | -0.097 | <0.001 |
| **Aortic valve calcification** | 0.006 | 0.31 |
| **Current smoking status** | 0.023 | 0.000 |
| **Current alcohol consumption status** | -0.002 | 0.757 |
| **hsCRP** | 0.053 | <0.001 |
| **Neutrophil absolute value** | 0.058 | <0.001 |
| **LDL-C** | 0.185 | <0.001 |
| **Prealbumin** | -0.010 | 0.091 |
| **Hcy** | -0.021 | <0.001 |
| **Uric acid** | -0.025 | <0.001 |
| **TC** | 0.113 | <0.001 |
| **Absolute lymphocyte value** | -0.008 | 0.157 |
| **TG** | -0.073 | <0.001 |
| **Albumin** | -0.027 | <0.001 |
| **Alkaline phosphatase** | -0.010 | 0.113 |
| **HbA1c** | 0.009 | 0.137 |
| **Red blood cell distribution width** | -0.012 | 0.041 |
| **Fibrinogen** | 0.162 | <0.001 |
| **Creatinine** | 0.017 | 0.005 |
| **eGFR** | -0.050 | <0.001 |
| **Fasting Glucose** | -0.037 | <0.001 |
| **Platelet count** | 0.134 | <0.001 |
| **BMI** | -0.068 | <0.001 |
| **ApoA** | 0.010 | 0.10 |
| **ApoB** | 0.136 | <0.001 |
| **HDL-C** | 0.076 | <0.001 |
| **SBP** | 0.013 | 0.026 |
| **DBP** | 0.000 | 0.951 |
| **NLR**  **PLR** | 0.054  0.111 | <0.001  <0.001 |

**ApoA** apolipoprotein A **ApoB** apolipoprotein B **BMI** body mass index **CHD** coronary heart disease **DBP** diastolic blood pressure **eGFR** glomerular filtration rate **hsCRP** high-sensitivity C-reactive protein **HbA1c** glycosylated hemoglobin **Hcy** homocysteine **HDL-C** high density lipoprotein cholesterol **LDL-C** low density lipoprotein cholesterol **SBP** systolic blood pressure **TC** total cholesterol **TG** triglyceride **T2DM** type 2 diabetes mellitus


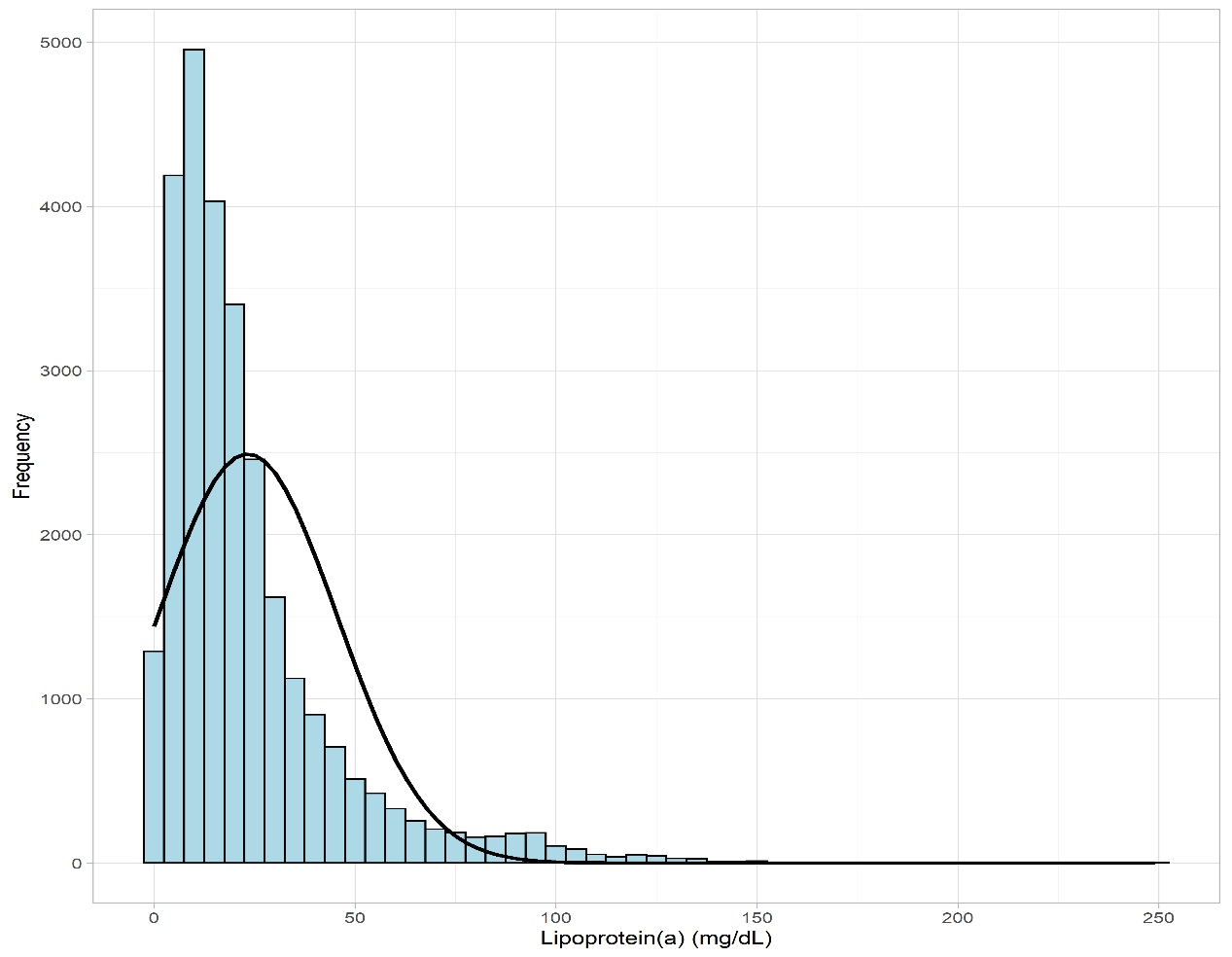


**Figure S1** Serum lipoprotein(a) level distribution of participants


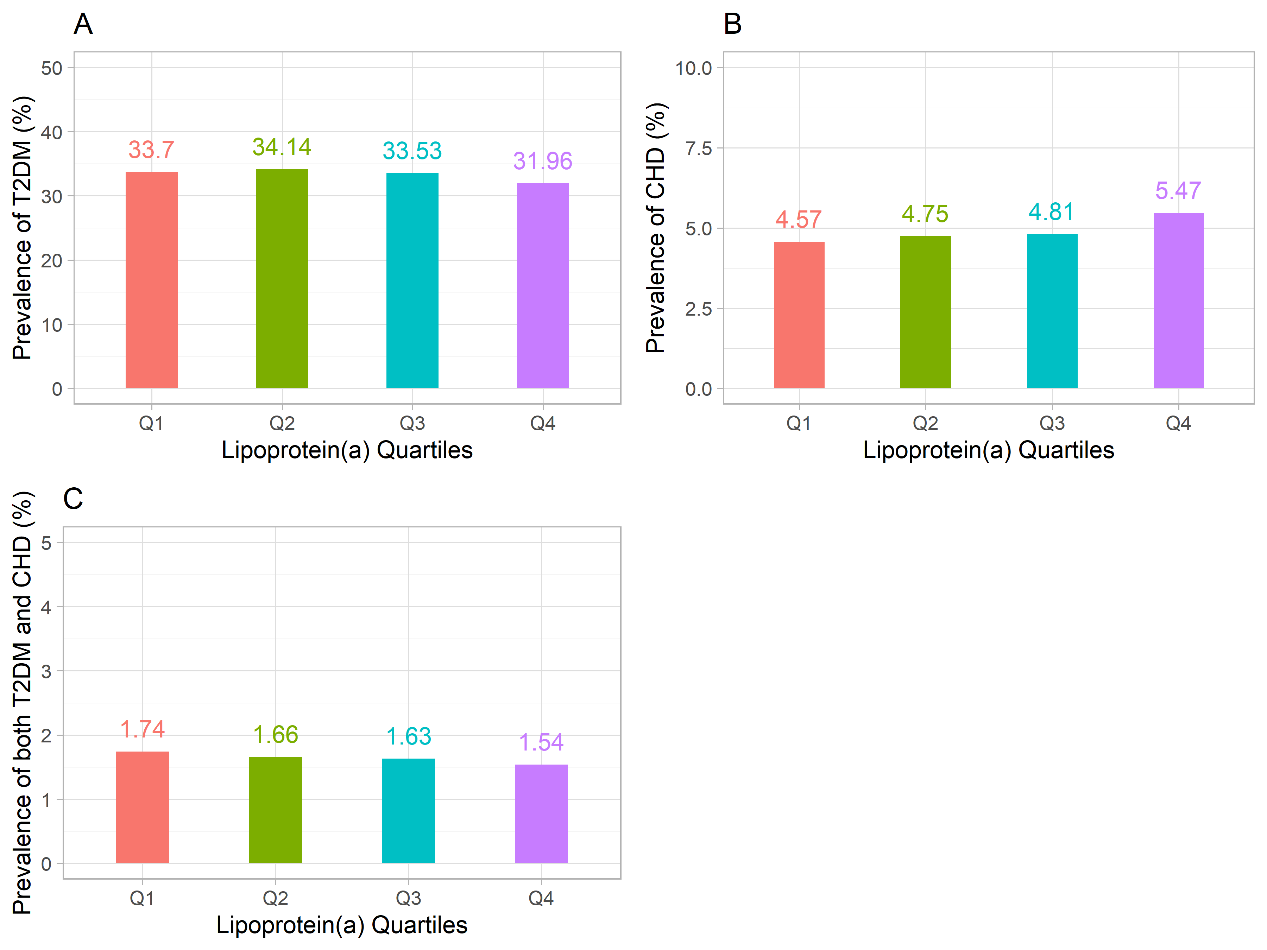


**Figure S2 Prevalence of T2DM and CHD according to quartiles of Lp(a) (mg/dl).** A. Prevalence of T2DM according to quartiles of Lp(a) (mg/dl). B. Prevalence of CHD according to quartiles of Lp(a) (mg/dl). C. Prevalence of T2DM with CHD according to quartiles of Lp(a) (mg/dl). The numbers above the bars are the prevalence of each outcome, respectively.
